# Sequential EBUS‐Guided Fine Needle Aspiration and Transbronchial Cryobiopsy for the Diagnosis of a Giant Mediastinal Lipoma: A Novel Intraoperative Pivot Strategy

**DOI:** 10.1002/rcr2.70633

**Published:** 2026-05-21

**Authors:** Venkatkiran Kanchustambham, Weston E. Bowker

**Affiliations:** ^1^ Department of Pulmonary and Critical Care Medicine Sanford Health Fargo North Dakota USA; ^2^ University of North Dakota School of Medicine and Health Sciences Fargo North Dakota USA

**Keywords:** cryobiopsy, endobronchial ultrasound, interventional pulmonology, MDM2 FISH, mediastinal lipoma

## Abstract

Mediastinal lipomas are extremely rare, comprising only 1.6%–2.3% of all primary mediastinal tumours, with fewer than 200 cases reported worldwide. The diagnostic imperative is excluding well‐differentiated liposarcoma via MDM2 fluorescence in situ hybridization (FISH), which has traditionally required surgical resection for adequate tissue sampling. We report the first described use of sequential EBUS‐guided fine needle aspiration (FNA) and intraoperative pivot to transbronchial cryobiopsy for definitive diagnosis of a giant mediastinal lipoma. A 61‐year‐old asymptomatic male was incidentally found to have a 6 × 7 × 13 cm anterior mediastinal mass on CT chest. EBUS‐FNA with a Cook 22‐gauge needle yielded only minute fat‐containing tissue on rapid on‐site evaluation (ROSE), insufficient for diagnosis. The procedure was immediately transitioned: an Olympus ViziShot 21‐gauge needle created a transbronchial tract through which a cryotherapy probe obtained multiple adequate tissue cores, confirming mature adipose tissue on histopathology. MDM2 FISH demonstrated no gene amplification, confirming benign lipoma. This case establishes a practical intraoperative rescue strategy for lipomatous mediastinal lesions where FNA yield is insufficient. The patient subsequently underwent surgical resection; final pathology confirmed benign lipoma, fully concordant with the bronchoscopic diagnosis.

## Graphical abstract

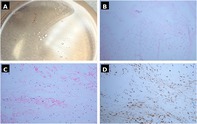

## 1 Introduction

Mediastinal lipomas are exceptionally rare benign tumours of mature adipocytes, accounting for only 1.6%–2.3% of all primary mediastinal tumours [1]. Fewer than 200 entirely intrathoracic lipomas have been reported in the world literature, and the vast majority arise asymptomatically in the anterior mediastinum, discovered incidentally on cross‐sectional imaging [2]. Their principal clinical significance lies in the imperative to exclude well‐differentiated liposarcoma (WDLS), a low‐grade malignancy that may appear radiographically similar and that carries substantially different surgical management implications [3].

Endobronchial ultrasound (EBUS) has transformed the minimally invasive evaluation of mediastinal pathology. Transbronchial cryobiopsy, in which a flexible cryoprobe is advanced beyond the bronchial wall under real‐time EBUS guidance, yields larger architecturally preserved tissue cores than conventional fine needle aspiration (FNA), potentially enabling histologic and molecular characterization of lesions previously requiring surgery [4]. We report the first case of sequential EBUS‐FNA and an intraoperative pivot to transbronchial cryobiopsy as a rescue strategy for the diagnosis of a giant mediastinal lipoma, with complete pathologic and molecular confirmation.

## 2 Case Report

A 61‐year‐old male financial advisor with a history of hypertension (managed with lisinopril) and Schatzki's ring presented to the emergency department on 2 February 2026, with intractable nausea and vomiting secondary to confirmed norovirus infection. He had no tobacco history, prior malignancy, pulmonary disease or significant occupational exposures. Functional status was excellent, with regular pickleball and treadmill exercise (> 4 METs).

**FIGURE 1:**
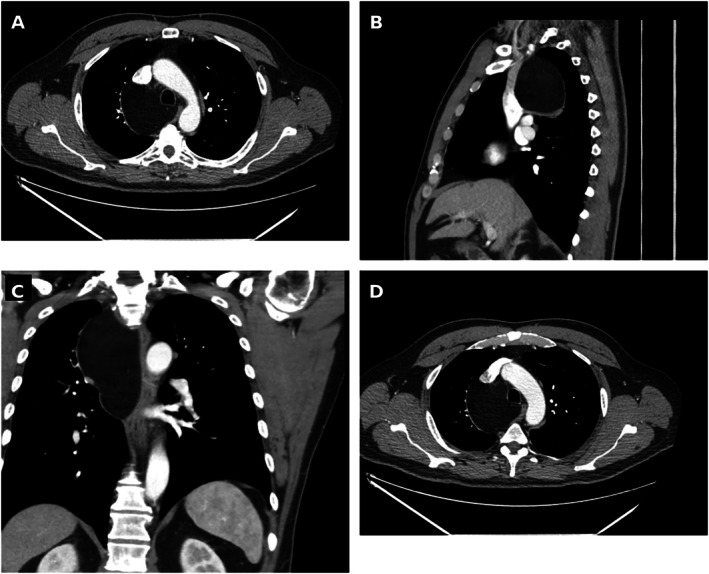
CT chest imaging demonstrating a giant anterior mediastinal lipoma. (A) Axial contrast‐enhanced CT at the level of the aortic arch showing a large anterior mediastinal mass with homogeneous fat attenuation (−100 HU), well‐defined smooth borders and no contrast enhancement. (B) Sagittal reconstruction demonstrating the full 13 cm craniocaudal extent of the mass from the thoracic inlet to the cardiac level. (C) Coronal reconstruction confirming the mass occupies the anterior mediastinum, displacing adjacent structures without invasion. (D) Axial CT at a more inferior level confirming homogeneous fat attenuation; the mass abuts the ascending aorta and main pulmonary artery without evidence of infiltration.

Abdominal and pelvic CT obtained during his admission identified a renal mass consistent with angiomyolipoma (AML). A subsequent chest radiograph revealed mediastinal widening, prompting CT of the chest. This demonstrated a 6 × 7 × 13 cm anterior mediastinal mass with homogeneous fat attenuation (approximately −100 HU), well‐defined smooth borders, and no internal nodularity, thick septations or contrast enhancement—features highly characteristic of a benign lipomatous lesion (Figure 1). Retrospective comparison with a cardiac screening CT from 2015 suggested the mass was either new or had enlarged over the intervening decade. The patient was entirely asymptomatic from the thoracic lesion.

He was referred to interventional pulmonology by cardiothoracic surgery for bronchoscopic tissue diagnosis prior to surgical planning. The procedure was performed under general anaesthesia. White‐light bronchoscopy demonstrated normal tracheobronchial mucosa and anatomy to at least the first subsegmental level, a sharp carina, mild clear secretions and no endobronchial lesions.

**FIGURE 2:**
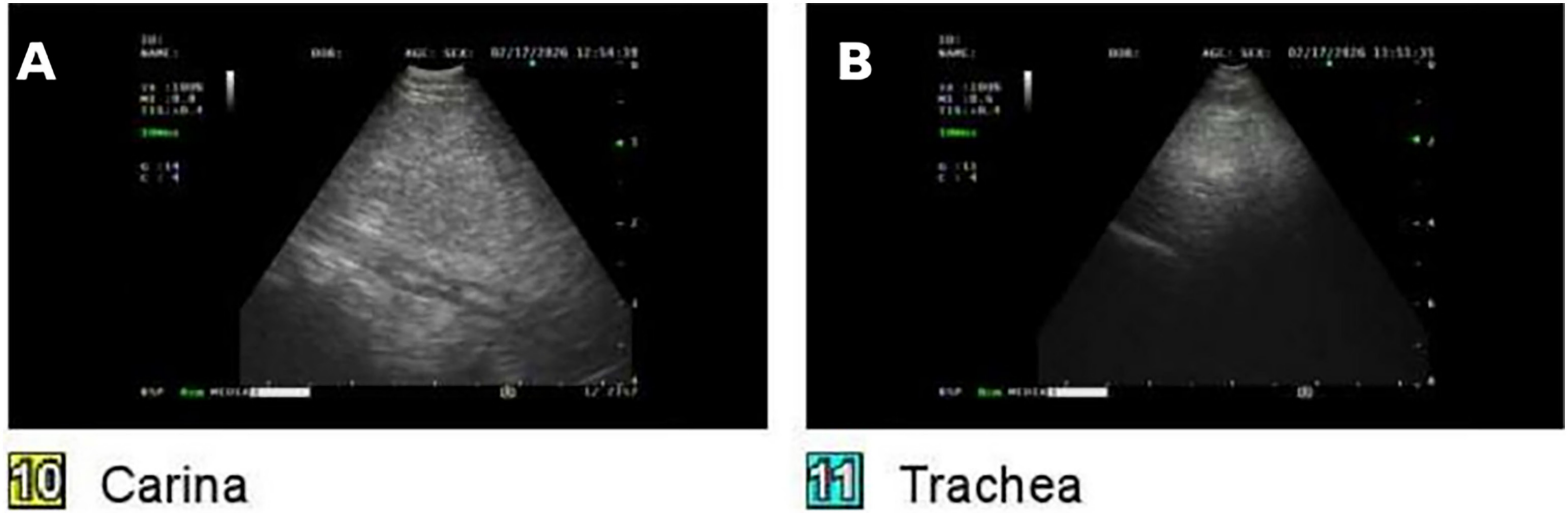
Intraoperative EBUS ultrasound images during bronchoscopic sampling (17 February 2026). (A) EBUS view at the level of the carina demonstrating the mediastinal mass as a homogeneous echogenic structure immediately adjacent to the airway wall, used to guide transbronchial tract creation and cryoprobe positioning. (B) EBUS view from the trachea confirming the superior extent of the mass under real‐time ultrasound visualization.

An EBUS endoscope was introduced and the mediastinal mass was identified under real‐time ultrasound guidance at the level of the carina and trachea (Figure 2). Because the sampling site was not visible endoscopically, a transbronchial technique was selected, with the sampling device penetrating the full thickness of the bronchial wall to access the mass. Initial sampling utilized a Cook 22‐gauge EBUS‐TBNA needle. Rapid on‐site evaluation (ROSE) confirmed fat‐containing tissue; however, FNA specimens yielded only a minute amount of material, insufficient for definitive histologic diagnosis or MDM2 FISH molecular testing.

**FIGURE 3:**
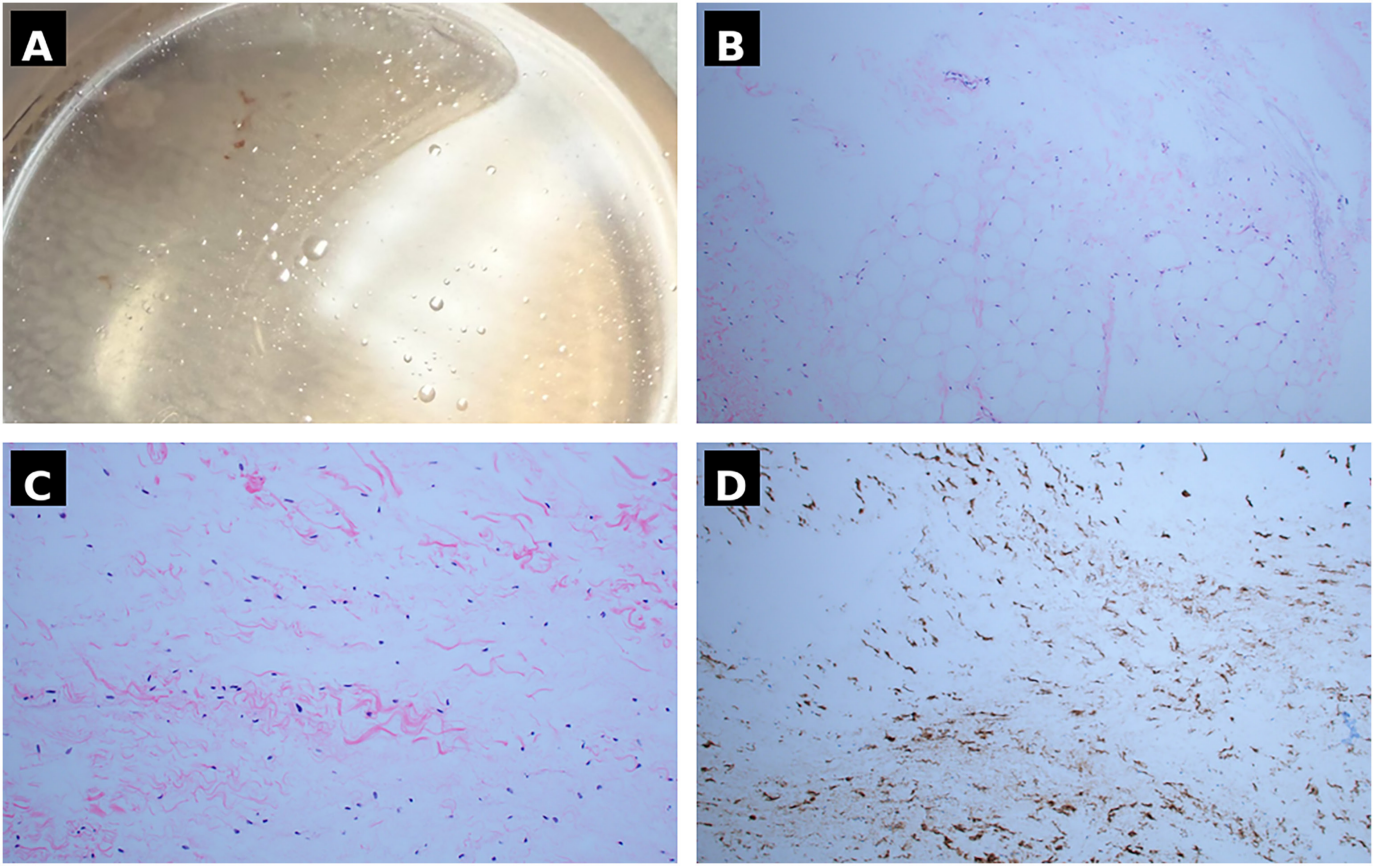
Pathologic characterization of transbronchial cryobiopsy specimens. (A) Gross specimen appearance demonstrating a characteristic oily supernatant upon saline immersion, providing immediate macroscopic confirmation of lipid‐rich tissue and correlating with ROSE findings of fat‐containing material. (B) Haematoxylin and eosin staining (low power) showing large, uniform lipid vacuoles with delicate fibrovascular septae and peripherally displaced nuclei, consistent with mature adipose tissue. (C) Haematoxylin and eosin staining (high power) confirming mature adipocytes without cytologic atypia, lipoblasts, nuclear pleomorphism or mitotic figures. (D) MDM2 fluorescence in situ hybridization (FISH) demonstrating non‐amplified signal patterns without MDM2 gene amplification, definitively excluding well‐differentiated liposarcoma and confirming the diagnosis of benign mediastinal lipoma.

Given inadequate FNA yield, the procedure was immediately transitioned intraoperatively to cryobiopsy. An Olympus ViziShot 21‐gauge needle was advanced to create a transbronchial tract into the mediastinal mass, followed by insertion of the needle sheath to maintain access. A flexible cryotherapy probe was then advanced through the established tract, and multiple transbronchial cryobiopsy specimens were obtained under continuous EBUS guidance. The gross appearance of the recovered specimens demonstrated a characteristic oily supernatant upon immersion, consistent with lipid‐rich tissue (Figure 3A). ROSE confirmed fat‐containing tissue and the procedure was completed without complications; no pneumothorax, clinically significant haemorrhage or hemodynamic instability occurred.

Final histopathology demonstrated mature adipose tissue with large, uniform lipid vacuoles and peripherally displaced nuclei, without cytologic atypia, lipoblasts, nuclear pleomorphism or mitotic figures (Figure 3B,C). MDM2 FISH demonstrated non‐amplified signal patterns without MDM2 gene amplification (Figure 3D), confirming the diagnosis of benign lipoma and definitively excluding well‐differentiated liposarcoma. The patient subsequently underwent elective thoracoscopic surgical resection by the cardiothoracic surgery team. Surgical pathology confirmed the diagnosis of benign mediastinal lipoma, fully concordant with the bronchoscopic diagnosis. The patient recovered without complication and is under joint follow‐up by interventional pulmonology and cardiothoracic surgery.

## 3 Discussion

This case reports, to our knowledge, the first diagnosis of a mediastinal lipoma via EBUS‐guided transbronchial cryobiopsy, and the first documentation of an intraoperative FNA‐to‐cryobiopsy pivot as a rescue strategy for lipomatous mediastinal lesions, with complete gross, histologic and molecular confirmation.

Mediastinal lipomas are extraordinarily rare. Mesenchymal tumours collectively constitute approximately 6% of all mediastinal masses, with pure lipomas representing a small minority [1]. A landmark historical compilation identified only 46 entirely intrathoracic lipomas in the world literature, with 35 surgical survivors [2]. Most arise asymptomatically in the anterior mediastinum and are detected incidentally, as in our patient, whose 13‐cm mass produced no cardiopulmonary symptoms despite its substantial size—consistent with prior reports of slow‐growing lesions with insidious mass effect [5].

Excluding WDLS is the central diagnostic imperative. CT features of homogeneous fat attenuation, smooth borders and absent enhancement favour lipoma, but are not pathognomonic; WDLS may demonstrate thick internal septa, nodularity or focal non‐fatty components [3]. MDM2 FISH is the definitive molecular discriminator, present in virtually all WDLS and absent in benign lipomas [6]. Obtaining tissue adequate for both histomorphology and FISH has historically mandated thoracic surgery or CT‐guided core needle biopsy. PET imaging was not performed in this case, a decision grounded in the recognized metabolic behaviour of lipomatous tumours. Well‐differentiated liposarcoma is characteristically FDG‐hypometabolic, and multiple studies have documented that PET lacks sufficient sensitivity and specificity to reliably distinguish benign lipoma from WDLS—both entities may appear PET‐negative. Therefore, tissue‐based molecular confirmation via MDM2 FISH analysis, which we obtained through the cryobiopsy specimen, represented the appropriate and definitive diagnostic strategy in this case.

The key procedural insight from this case is that ROSE‐confirmed fat with inadequate FNA volume should serve as a recognized intraoperative trigger for cryobiopsy escalation. Adipose tissue is cohesive by nature and resists needle aspiration regardless of positioning accuracy—making FNA yield failure predictable for lipomatous lesions. Crucially, while ROSE confirmed adipose tissue, cytologic smear preparations from FNA are insufficient substrate for MDM2 FISH analysis, which requires architecturally preserved tissue cores with adequate cellularity. The intraoperative escalation to cryobiopsy was therefore driven not by diagnostic uncertainty about tissue type, but by the imperative to obtain a specimen adequate for molecular testing—the only definitive means of excluding WDLS. Additionally, the bronchoscopic approach simultaneously allowed assessment of mediastinal lymph nodes for involvement, providing staging‐relevant information within the same anaesthetic event. The characteristic oily supernatant visible on gross specimen examination provided immediate macroscopic confirmation of lipid‐rich tissue, further supporting the intraoperative decision to escalate. Cryoadhesion retrieves tissue en bloc, overcoming the fundamental limitation of aspiration‐based techniques. The use of the Olympus ViziShot 21‐gauge needle to pre‐form the transbronchial tract prior to cryoprobe insertion provided stable access while maintaining real‐time EBUS visualization. Critically, the entire escalation occurred within a single bronchoscopic session, avoiding the morbidity of a second procedure or thoracic surgery.

The synchronous renal AML finding is of incidental interest. Both mediastinal lipoma and renal AML are benign mesenchymal adipocytic tumours; renal AML is a hallmark of tuberous sclerosis complex (TSC), though sporadic cases predominate. In the absence of other TSC stigmata in our patient, coincidental co‐occurrence is most likely. Clinicians encountering this combination should nonetheless consider clinical evaluation for TSC‐related features.

In conclusion, EBUS‐guided transbronchial cryobiopsy, deployed as an intraoperative rescue following FNA failure, represents a safe, effective and reproducible minimally invasive strategy for pre‐surgical diagnosis of mediastinal lipomatous lesions. In this case, the bronchoscopic approach enabled definitive tissue and molecular diagnosis, lymph node assessment and informed surgical planning—all within a single anaesthetic event—prior to the patient's subsequent elective surgical resection, which confirmed a benign lipoma. Recognition of ROSE‐confirmed fat with inadequate FNA volume as a cryobiopsy trigger, supported by characteristic gross lipid appearance, represents a reproducible intraoperative decision framework for this rare but diagnostically challenging entity. Whether this approach may obviate surgery in select cases warrants evaluation in future prospective series.

## Author Contributions

V.K. conceived the case report, performed the bronchoscopic procedure and drafted the manuscript. W.E.B. performed the pre‐procedural clinical evaluation and contributed to manuscript revision. Both authors reviewed and approved the final manuscript.

## Consent

The authors declare that written informed consent was obtained for the publication of this manuscript and accompanying images using the form provided by the Journal.

## Conflicts of Interest

V.K. is an Editorial Board member of Respirology Case Reports and a co‐author of this article. He was excluded from all editorial decision‐making related to the acceptance of this article for publication. The authors declare no conflicts of interest.

## Data Availability

The data that support the findings of this study are available on request from the corresponding author. The data are not publicly available due to privacy or ethical restrictions.
